# Odanacatib Coating Supports Osseointegration of Implants: A Preclinical Study

**DOI:** 10.1111/clr.70038

**Published:** 2025-09-04

**Authors:** Natália dos Santos Sanches, Caroline Liberato Marchiolli, Lara Cristina Cunha Cervantes, Maria Cristina Ruiz Voms Stein, Sara Alves Berton, Estéfany Lopes Lemes do Prado, Carina Kampleitner, Francisley Ávila Souza, Roberta Okamoto, Reinhard Gruber, Idelmo Rangel Garcia Júnior

**Affiliations:** ^1^ Department of Diagnosis and Surgery São Paulo State University (UNESP), School of Dentistry Araçatuba São Paulo Brazil; ^2^ Department of Oral Biology University Clinic of Dentistry, Medical University of Vienna Vienna Austria; ^3^ University of Brazil São Paulo Brazil; ^4^ Karl Donath Laboratory for Hard Tissue and Biomaterial Research University Clinic of Dentistry, Medical University of Vienna Vienna Austria; ^5^ Austrian Cluster for Tissue Regeneration Vienna Austria; ^6^ Ludwig Boltzmann Institute for Traumatology The Research Center in Cooperation With AUVA Vienna Austria; ^7^ Department of Basic Sciences São Paulo State University (UNESP), School of Dentistry Araçatuba São Paulo Brazil; ^8^ Department of Periodontology, School of Dental Medicine University of Bern Bern Switzerland

**Keywords:** μCT, biomechanical testing, dental implants, histomorphometry, odanacatib, osseointegration, rat

## Abstract

**Introduction:**

Odanacatib (ODN), a cathepsin K inhibitor, is a drug that reduces bone resorption while preserving bone formation. ODN was initially developed for the treatment of postmenopausal osteoporosis, but further development as a systemic medication has been discontinued. Here, we propose ODN as a topical treatment, the coating of dental implants, to achieve an anabolic shift of early osseointegration.

**Material and Methods:**

To this aim, we have coated double acid etching titanium implants (SLA) with and without ODN dispersed in a simulated body fluid (SBF). The implants were inserted into the tibia of 72 male Wistar rats and osseointegration was studied on Days 15 and 40. Biomechanical testing, micro‐computed microtomography, and histomorphometric analyses were performed.

**Results:**

Biomechanical testing reveals that after 15 days of healing, removal torque increased from 2.5 Ncm (1.5–4.4) to 3.9 Ncm (1.7–7.6) when comparing SBF alone with SBF containing ODN, respectively (*p* = 0.017). Consistently, micro‐computed microtomography indicated that local bone volume increased from 24.4% (13.86–32.45) to 32.8% (19.7–39.4), respectively (*p* = 0.003). The same was true for presumed bone‐to‐implant contact, which was 34.38% (27.0–50.0) and 43.33% (31.6–54.2), respectively (*p* = 0.035). Histomorphometric analyses confirmed that the new bone area per total area increased from 34.78% (18.9–43.7) to 41.10% (23.5–54.0), respectively (*p* = 0.102). This trend proceeds after 40 days of osseointegration.

**Discussion:**

These findings suggest that topical delivery of a cathepsin K inhibitor can support the early osseointegration phase in an ectopic rodent implantation model.

## 1 Introduction

Implant dentistry became an established therapy to replace missing teeth, allowing patients to return to their original chewing comfort. While titanium implants originally had a polished or machined surface, today, implants are equipped with a moderately rough surface that can be obtained by acid etching, sandblasting, or anodization to improve their osteophilic properties (Bosshardt et al. 2017). Implant surfaces allow new bone to firmly attach to the implant surface, providing stable support for its loading. This process is traditionally termed osseointegration (Brånemark et al. 1977). It is characterized by a catabolic phase where bone‐resorbing osteoclasts dominate the scene, followed by an anabolic phase where bone‐forming osteoblasts repopulate the small peri‐implant bone defects (Berglundh et al. 2003). The newly formed bone bridges the small gap between the pristine bone and the implant surface (Berglundh et al. 2003; Vasak et al. 2014). Among the main challenges in implant dentistry is to keep the transition from the catabolic to the anabolic phase short (Smeets et al. 2016), particularly when we follow the concept of early or immediate implant loading (Morton et al. 2023). Here we propose a topical pharmacological strategy that supposedly reduces osteoclast activity while maintaining the function of osteoblasts to boost the natural sequence of osseointegration even further.

Previous strategies aiming to reduce osteoclast activity were based on coating dental implants (Abtahi et al. 2019; Mokhtari et al. 2024) and orthopedic rods (Tanzer et al. 2005; Zhang et al. 2023) with bisphosphonates, commonly used antiresorptive drugs to reduce the risk of osteoporotic fractures (Favus 2010; Reid and Billington 2022). Bisphosphonates are also applied in oncology to lower skeletal‐related events such as hypercalcemia and concomitant excessive bone loss increasing fracture risk (D'Oronzo et al. 2021). Bisphosphonates have no deleterious effect on fracture healing likely because in early fracture healing, bone formation is not dependent on its previous resorption (Chandran et al. 2024). In regular bone remodeling, however, bisphosphonates lower bone resorption but, because of coupling (Sims and Martin 2020), also bone formation is reduced (Black et al. 2007). Even though coating with bisphosphonates increased osseointegration and implant stability, concerns have been raised about the ability of bisphosphonates to absorb to bone and cause a long‐term decrease in bone remodeling, a process with an unclear clinical impact (Ashrafi et al. 2021). Apart from their beneficial effects in reducing bone fractures, osteonecrosis was identified as a rare but significant side effect of bisphosphonate therapy (Khosla et al. 2007; Anastasilakis et al. 2022). Other pharmacological osteoporosis therapies follow a different principle; teriparatide, for instance, a truncated form of parathyroid hormone with a bone anabolic activity, also supports osseointegration (Kuchler et al. 2011) and accelerated osseous wound healing in the oral cavity (Bashutski et al. 2010); however, only upon daily injection.

Odanacatib, an inhibitor of the main collagenase in osteoclasts, cathepsin K (Dai et al. 2020), was initially considered a revolution in osteoporosis therapy (Chen et al. 2023) because the pharmacological mechanism differs from that of bisphosphonates and other antiresorptive agents. Support for this new paradigm is based on its ability to reduce osteoclast activity without killing the cells or blocking the process of osteoclastogenesis (Chapurlat 2015; dos Santos Sanches et al. 2025)—and thus allows the non‐resorbing but vital osteoclasts to signal to the bone‐forming osteoblast. Consequently, osteoblast activity is maintained, even though osteoclast activity is reduced—which contrasts with the bisphosphonates, which, at least when applied systemically, lower both resorption and formation. Odanacatib, however, was stopped at phase III fracture trials because of an increased risk of stroke, even though the bone mineral density risk of fractures was reduced (McClung et al. 2019). A similar destiny has reached several other cathepsin inhibitors, such as balicatib and relacatib. Currently, ODN is classified as an experimental drug with no approved legal status for clinical or commercial applications worldwide. Even though ODN was supposed to control dentin erosion (Chen et al. 2023) and prevent bone loss during the progression of periodontitis (Hao, Chen, McConnell, et al. 2015; Hao, Chen, Zhu, et al. 2015), this research remains in the experimental stage. Hence, accumulating evidence has led to a closing of the chapter on systemic treatment of metabolic bone diseases with cathepsin K inhibitors; however, no attempts were made to benefit from the function of ODN in opening a new chapter of a topical rather than a systemic approach. For instance, research on titanium discs provided valuable insights that demonstrated that the cathepsin K activity of the osteoclast lysate was reduced in the presence of ODN‐coating and did not affect osteoclastogenesis or osteoclast survival (dos Santos Sanches et al. 2025).

The present study aims to test this novel concept of coating implants with ODN based on the hypothesis that a simultaneous blocking of bone resorption while, at the same time, supporting or at least not hindering bone formation supports osseointegration. In addition, since ODN has a transient activity, the long‐term risk of suppressed bone remodeling around dental implants, and thus the ability of the bone to undergo modeling according to Wolf's law (Frost 2003), can almost be ruled out. There is, therefore, a strong rationale to assume that implants coated with this cathepsin K inhibitor show more local bone formation than the respective control implants and that this beneficial activity is limited to the early stages of osseointegration. We have tested this hypothesis in a rat tibia implantation model, and our findings support the original hypothesis, as indicated in the present report.

## 2 Materials and Methods

### 2.1 Experimental Animals

**FIGURE 1:**
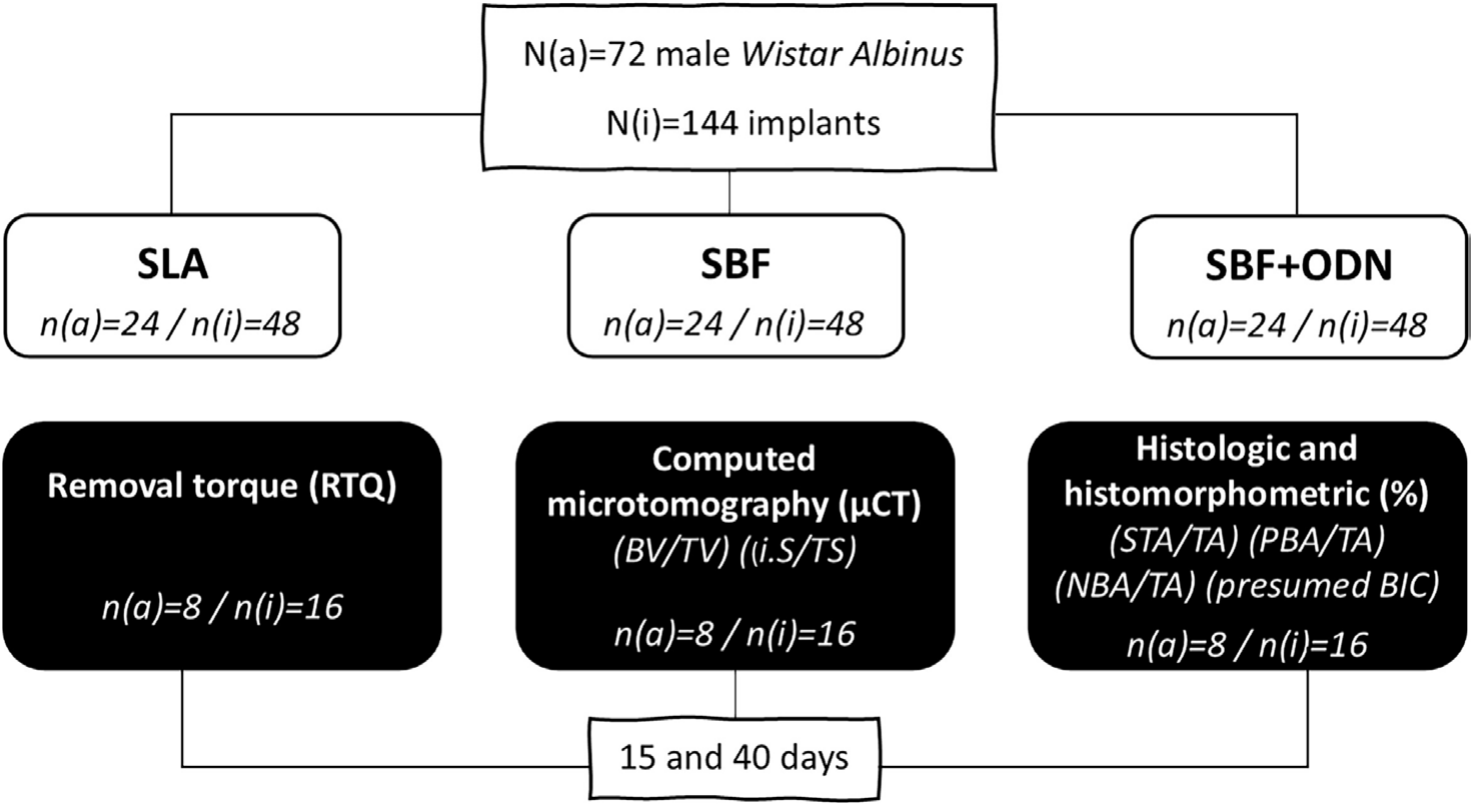
The workflow illustrates the distribution of samples into three implant surface treatment groups: SLA, SBF (control), and SBF + ODN. N(a) represents the number of animals per group, while N(i) represents the number of implants analyzed. The experimental design followed *n* = 8 animals/group/period. The diagram describes the analyses performed and the evaluation periods of 15 and 40 days.

This research project was approved by the Ethics Committee on the Use of Animals (CEUA: 2021‐524 and 0431‐2022). The reported data conform to ARRIVE (Animal Research: Reporting of In Vivo Experiments) guidelines and follow the National Institutes of Health Guide for the Care and Use of Laboratory Animals. The sample size was estimated based on unpublished observations, ensuring a significance level of 5% and a statistical power of 80%, and based on a previous study considering (*n* = 8 animals/group/period) (Ramalho‐Ferreira et al. 2015; Inoue et al. 2023). This study involved 72 male Wistar Albinus rats, aged 3 months, weighing 350 and 450 g. Rats were housed in ventilated cages with a 12/12‐h light/dark cycle and received food and water ad libitum. Animals were acclimated for 1 week before being randomly assigned to one of three treatments. The allocation of animals was carried out by a blinded experimenter using the random.org tool. The treatment groups were as follows: (1) implant with double acid etching surface treatment (SLA), (2) implant with double acid etching and simulated body fluid (SBF), (3) implant with treatment by double acid etching, simulated body fluid, and odanacatib (MedChemExpress LLC, Monmouth Junction, NJ) at a concentration of 260 μg/mL (SBF + ODN). The rats were sacrificed after 15 and 40 days for further analysis (Figure 1). The concentration of ODN for the implant coating was determined based on preclinical studies involving systemic administration of 30 mg/kg (Yi et al. 2017). Local release calculations were performed to achieve a therapeutic dose of 10.5 mg of ODN over a 4‐day biomimetic treatment protocol (Queiroz et al. 2013; dos Santos Sanches et al. 2025). As a result, a concentration of 260 μg/mL in SBF was selected to ensure controlled and effective drug release while maintaining safety. Furthermore, data from mouse models demonstrated that a weekly systemic dose of 3.6 mg/kg (Hao, Chen, McConnell, et al. 2015) for ODN corresponds to an approximate equivalent dose of 1.3 mg for a 350 g rat. This supports the appropriateness of the chosen 260 μg/mL concentration, which is well within the range necessary to achieve localized therapeutic efficacy in a controlled‐release context.

### 2.2 Coating of Implants

The manufacturer carried out SLA treatment of implants (1.6 mm diameter, 3.0 mm length; Implalife, Medical‐Dental Products Industry, Jales, São Paulo, Brazil) following a patented protocol. Next, implants underwent surface coating with SBF, both with and without ODN at a concentration of 260 μg/mL (SBF + ODN), as described recently (Queiroz et al. 2013; dos Santos Sanches et al. 2025). The implants were first immersed in 5.0 mol/L NaOH (Sigma‐Aldrich, St. Louis, MO, USA), ensuring complete submersion of the threads, and incubated at 60°C for 24 h to activate their surfaces. They were then dried at the same temperature for an additional 24 h. Following this, the implants were immersed in SBF (pH 7.25) at 37°C for 4 days, either with or without ODN. To maintain ionic balance and ensure solution stability, the SBF solutions were refreshed daily throughout the incubation. Afterward, the implants were dried at 60°C for 24 h. The next step involved washing the implants in a 10% hydrogen peroxide (Sigma‐Aldrich) at room temperature for 4 h, with the solution replaced hourly. This was followed by a brief rinse in 0.9% sodium chloride solution and absolute ethanol. Finally, the implants were thoroughly dried, individually packaged, and gamma‐sterilized (Implalife). The implant coating was evaluated using scanning electron microscopy (SEM; Evo LS15, Zeiss, Jena, Germany) coupled with an energy‐dispersive X‐ray spectroscopy (EDX) system for semi‐quantitative analysis of chemical composition and elemental mapping on the surfaces.

### 2.3 Implant Insertion and Topographic Characterization of Surfaces

Animals received an intraperitoneal injection of 10% ketamine hydrochloride at 100 mg/kg (Cetamin) and xylazine at 5 mg/kg (Xilazin, both Petsupermarket Trade of Animal Products Ltd., São Paulo, Brazil). Following the surgical approach, as described recently (Ramalho‐Ferreira et al. 2015; Faverani et al. 2018), an incision was made in the proximal metaphysis of the left tibia, and the muscles and periosteum were displaced. A 1.4 mm diameter hole was drilled (Implalife, Medical‐Dental Products Industry, Jales, São Paulo, Brazil), approximately 5 mm distal to the physis in the metaphyseal region, using a reduction ratio of 16:1, at a speed of 1000 rpm, to a depth of 3.0 mm. Implants were inserted using a torque wrench for 1.3 mm square connections, secured with bicortical locking. Each tibia received one implant. The wounds were closed with absorbable sutures (Vicryl 4‐0; Ethicon GmbH). Postoperatively, a single intramuscular dose of 0.2 mL veterinary antibiotic (Pentabiotic for small animals, Fort Dodge Animal Health Ltda., Campinas, Brazil) and sodium dipyrone (1 mg/kg/day; Ariston Chemical and Pharmaceutical Industries Ltda., São Paulo, Brazil) was administered intramuscularly. The animals were euthanized after 15 and 40 days using an overdose of Cetamin and Xilazin.

### 2.4 Biomechanical by Removal Torque (RTQ)

The biomechanical analysis was performed by removing the implants using a digital torque meter (Lutron TQ‐8800; Lutron Instruments, London, United Kingdom). A counterclockwise movement was applied until the implant rotated within the bone tissue, causing a complete rupture of the bone‐to‐implant interface. The torque meter recorded the maximum rupture value in Newton centimeters (Ncm).

### 2.5 Computed Microtomography

**FIGURE 2:**
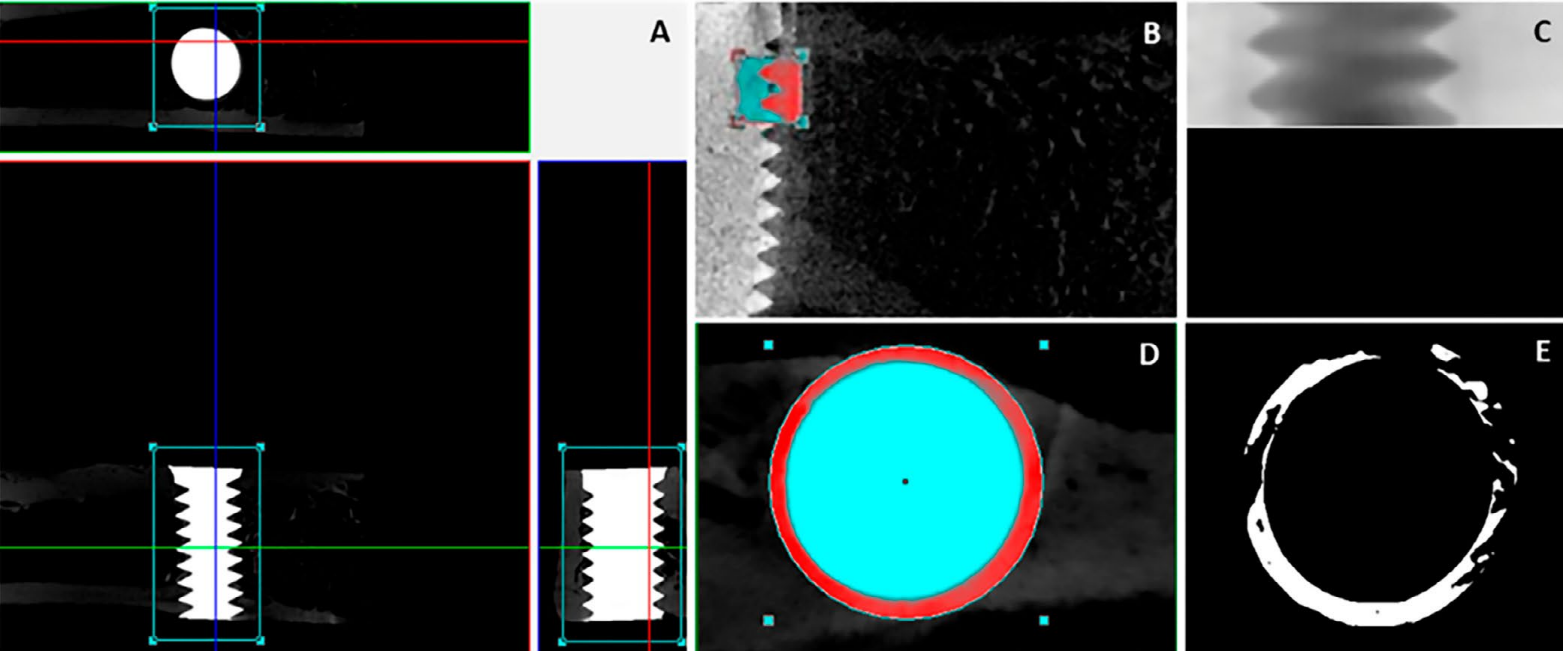
Micro‐CT analysis of the implants was performed after sample reconstruction and the selection of a standard position in the Data Viewer (A) software. The red frame highlights the area of interest between the first and third implant threads (B), while the circular region indicates the defined ROI of 1.95 × 1.95 mm as measured in the CTAn software (D). The VOI is shown after processing in the CTAn (C), illustrating the ROI that includes only bone tissue (E).

Samples were scanned by the SkyScan 1272 CMOS—high‐resolution XRM (Bruker MicroCT, Kontich, Belgium) using 9 μm thick cuts (50 kV and 500 μ), with a copper and aluminum filter and 0.3 mm rotation pitch. The images obtained from X‐ray projections were stored and reconstituted, with the area of interest defined using NRecon v.1.6.6.0 (Bruker Corporation, Billerica, MA). The Data Viewer software v.1.4.4 64‐bit (SkyScan, Leuven, Belgium) aligned all samples in a standard position, allowing observation in three planes: transverse, longitudinal, and sagittal (Figure 2A–C). The coronal view and the specific area of interest were then selected for standardizing the region of interest (ROIs) by 1.95 × 1.95 mm (Figure 2D) in 90 slices from the first to the third thread of the implant—the volume of interest (VOI) (Figure 2D,E) was delimited for the trabecular bone within the medullary compartment. The area for three‐dimensional evaluation was defined using the CTAn software v.1.18. (Bruker Corporation, Billerica, MA). The images were converted to grayscale with a range from 0 to 255, with the minimum threshold set at 50 and the maximum at 125. They were providing sufficient grayscales for all groups to carry out the three‐dimensional morphometry for the percentage of bone volume per tissue volume (BV/TV) following the guidelines of the Bruker manual. The percent intersection surface (i.S/TS%) was calculated using 2D morphometric analysis, excluding the cut ends of the VOI to avoid artificial overestimation of bone contact. The peripheral VOI surface (TS%) was used as the denominator, ensuring an accurate representation of the bone‐implant contact (BIC).

### 2.6 Hard Tissue Processing and Analysis

Samples decalcified in 20% EDTA for 6 weeks were paraffin‐embedded. For implant removal during the paraffin embedding process, we inverted the specimen, positioning the upper part of the implant facing downward in contact with the mold. Once the paraffin had completely solidified, we carefully melted the paraffin in contact with the implant, ensuring that the surrounding bone tissue remained unaffected. Next, we inserted the square digital key and carefully removed the implant. After the implant was fully removed, we repeated the paraffin bath to ensure complete incorporation, avoiding bubbles and preserving the threads of the dental implant. The re‐embedding step was performed to ensure optimal preservation of the surrounding bone tissue and prevent artifacts (Figure S1). Subsequently, histological slides were prepared, allowing the coronal view of the implants. Longitudinal sections to the implant axis were stained with hematoxylin and eosin. This technique enables histological sections without compromising the integrity of the implant threads. Slides were scanned with a digital microscope (MoticEasyScan One Scanner, Hong Kong, China). Histomorphometric analyses were conducted using Adobe Photoshop CS6 (Adobe Inc., San Jose, CA), with a 104 μm/400 pixels extension to the implant surface and ImageJ software (Schneider et al. 2012)—within 104 μm/400 pixels extension adjacent to the implant surface. The ROI was set within the most coronal and apical bone‐to‐implant contact. Respective areas of newly formed bone (NB), pristine bone (OB), and soft tissue (ST) were manually segmented and quantified, and corresponding percentages were calculated as a ratio to the total tissue area (TA). Data were reported using the following abbreviations: new bone area per tissue area (NBA/TA), pristine bone area per tissue area (PBA/TA), and soft tissue area per tissue area (STA/TA). Further, the implant surface in contact with mineralized bone, bone‐to‐implant contact (BIC), was measured, under the restriction that implants were carefully removed during the paraffin‐embedding process and re‐embedded to prepare histological slides, making this a presumed BIC.

### 2.7 Statistics

Statistical analyses were conducted using GraphPad Prism 8.0.1 (GraphPad Software, Boston, MA). A mixed‐effects model (REML) for one‐way ANOVA with Geisser–Greenhouse correction was performed, followed by Dunnett's test for multiple comparisons. The analyses were applied to the biomechanical values, percentages of bone volume—(BV/TV), intersection surface (i.S/TS), new bone area (NBA/TA), pristine bone (PBA/TA), soft tissue (STA/TA), and presumed bone‐to‐implant contacts (BIC). The *p*‐values are expressed in the figures.

## 3 Results

### 3.1 Quality Control of Surface Coating

**FIGURE 3:**
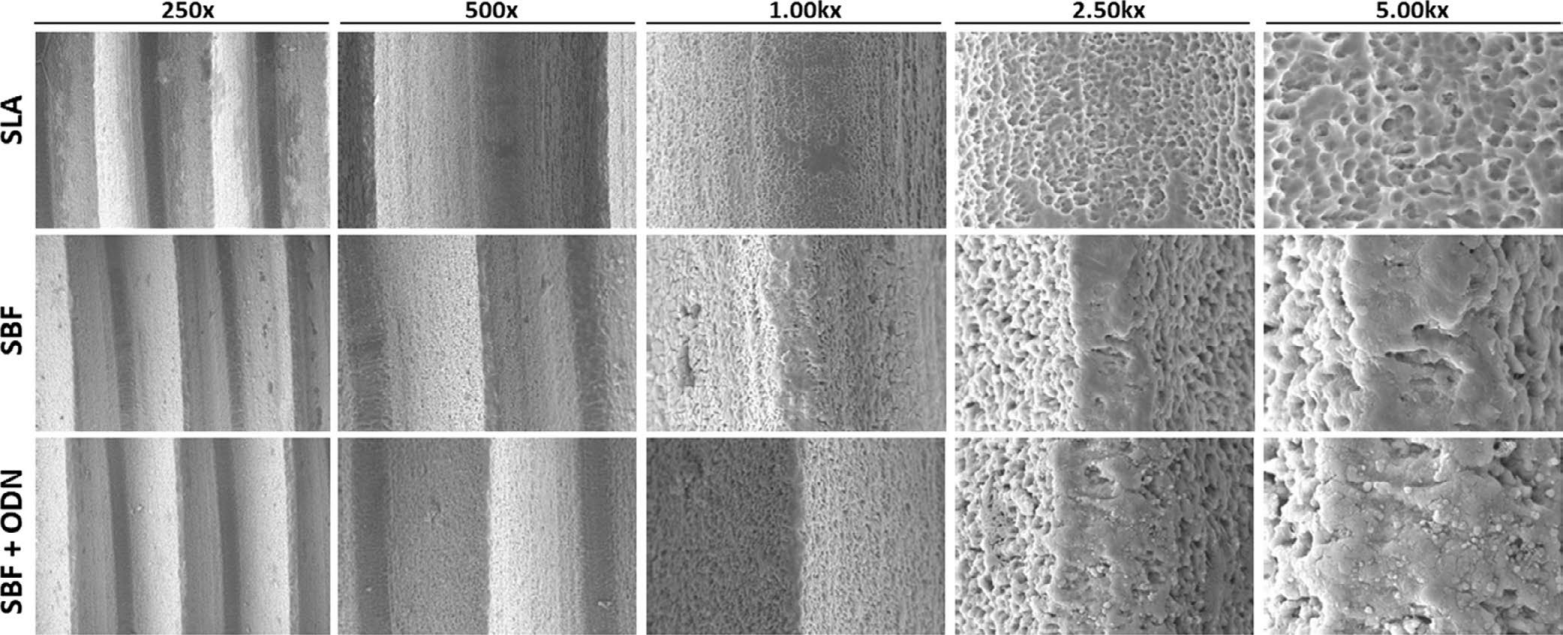
Scanning electron microscopy (SEM) images were obtained for SLA, SBF, and SBF + ODN. SLA exhibited an evenly rough texture with smaller pores. SBF surface demonstrated a uniform roughness, characterized by larger, globular, and interconnected pores. Similarly, the SBF + ODN group revealed a uniformly rough texture with larger, globular, and interconnected pores. At higher magnifications, nanometric particle deposition was evident in the SBF and SBF + ODN groups.

**FIGURE 4:**
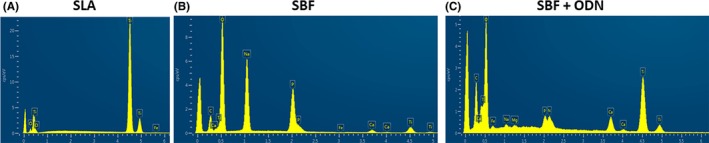
Energy‐dispersive X‐ray (EDX) spectroscopy of SLA, SBF, and SBF + ODN implant surfaces with an iron (Fe) coating for analysis. (A) The SLA surface shows prominent titanium (Ti) peaks. (B) The SBF surface presents calcium (Ca) and phosphorus (P) peaks. (C) The SBF + ODN surface exhibits peaks for carbon (C), nitrogen (N), and oxygen (O), associated with ODN, along with calcium and phosphorus peaks.

The coating of dental implants with SBF is widely used for delivering growth factors and other molecules to the defect site (Kodama et al. 2008; Wu et al. 2008). SBF coating requires confirmation by scanning electron microscopy. Our topographic analysis revealed that SLA coating exhibited a typical subtractive pattern, characterized by microcavities of varying depths and sizes, maintaining a homogeneous roughness (Figure 3). SBF treatment produced a surface with globular and interconnected pores featuring a hydroxyapatite cobbled nanometric appearance, resembling “cauliflower” (Bellucci et al. 2019). The addition of ODN to SBF resulted in a moderate increase in surface irregularity, indicated by a random distribution of smaller hydroxyapatite particles (dos Santos Sanches et al. 2025; Figure 3). The EDX analysis confirmed the absence of contamination on the titanium implant surfaces. As expected, the SLA treatment exhibited titanium (Ti) peaks, along with the presence of oxygen (O). The SBF treatment alone showed calcium (Ca) and phosphorus (P) peaks, indicating the deposition of calcium phosphate (CaP) (Queiroz et al. 2013; Santos et al. 2023). In the SBF + ODN group, carbon (C), nitrogen (N), and oxygen (O) were detected, corresponding to the ODN components, alongside Ca and P signals, though with lower intensity (dos Santos Sanches et al. 2025; Figure 4).

### 3.2 Removal Torque (RTQ) of Integrated Implants

**FIGURE 5:**
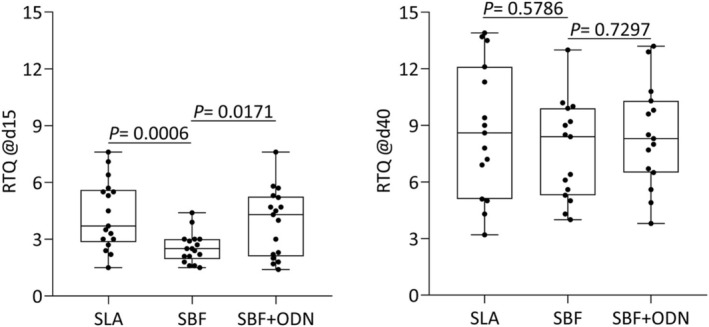
Removal torque (RTQ) expressed by the peak Ncm of SLA, SBF, and SBF + ODN allowed to heal for 15 and 40 days. At 15 days, significant differences were observed comparing SLA and SBF groups (*p* = 0.0006), and SBF and SBF + ODN groups (*p* = 0.0171). At 40 days, no statistical difference was observed between the groups.

Considering the clinical relevance of mechanical bone support, we evaluated the torque needed to unscrew the implants. Less RTQ was needed to unscrew implants on Day 15 than on Day 40 (Figure 5). Odanacatib caused a significant increase in RTQ at Day 15 when compared to SBF‐alone implants (*p* = 0.0171)—an advantage that diminished during the later observation period. However, when compared to SBF, uncoated SLA implants are more stably anchored in the bone at Day 15 (*p* = 0.0006) (Figure 5; Table 1).

**TABLE 1:** RTQ expressed by the peak values in Ncm, the mean, and the standard deviation (±) of the samples for each group: SLA, SBF, and SBF + ODN in periods of 15 and 40 days.

Groups	SLA	SBF	SBF + ODN	*p*
15 days	3.70 ± 1.82	2.50 ± 0.79	4.30 ± 1.79	*p* = 0.0006; *p* = 0.0171
40 days	8.60 ± 3.55	8.40 ± 2.63	8.30 ± 2.73	*p* = 0.5786; *p* = 0.7297

*Note:* A mixed‐effects model (REML) for one‐way ANOVA with Geisser–Greenhouse correction was performed, followed by Dunnett's test for multiple comparisons.

### 3.3 Micro‐CT Analysis to Determine Peri‐Implant Bone Volume

**FIGURE 6:**
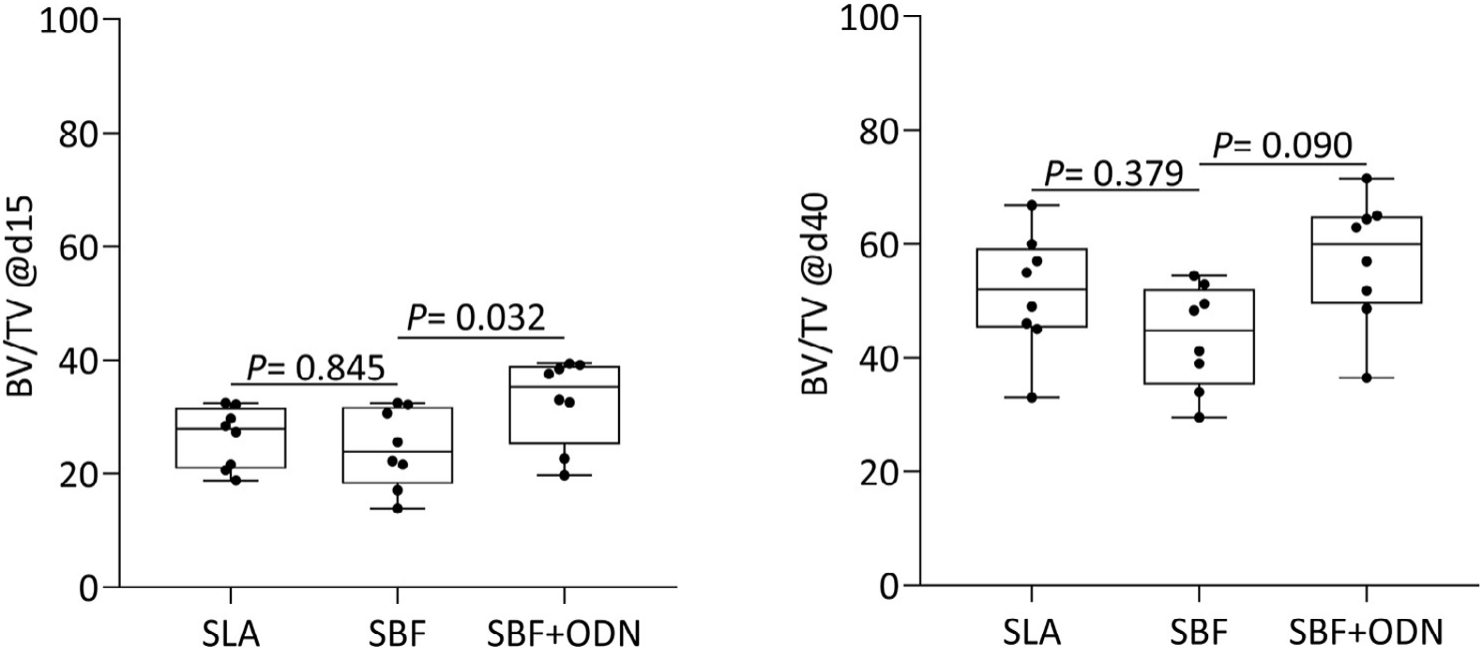
Micro‐CT analysis of the implants was performed considering the 1.95 × 1.95 mm regions of interest (ROIs). Graphs representing the percentage of bone volume per tissue volume (BV/TV) for each group: SLA, SBF, and SBF + ODN at Day 15 and Day 40. Significant differences were observed comparing the groups SBF and SBF + ODN at Day 15 (*p* = 0.032) and Day 40 (*p* = 0.090).

We then investigated the peri‐implant bone volume by μCT analysis. Consistent with the removal torque values, there was a gain in radiological BV/TV between Day 15 and Day 40 (Figure 6; Table 2). The presence of ODN increased the peri‐implant bone volume over SFB alone, not only on Day 15 but also on Day 40 (Figure 6), as well as i.S/TS on Day 15 (Table 2). Again, SBF coating was less favorable than uncoated SLA surface implants.

**TABLE 2:** Micro‐CT 2D morphometric analysis of the percent intersection surface (i.S/TS%), the mean, and the standard deviation (±) of the samples for each group: SLA, SBF, and SBF + ODN.

Groups	SLA	SBF	SBF + ODN	*p*
15 days	29.5 ± 6.7	28.1 ± 8.4	34.9 ± 3.7	*p* = 0.79; *p* = 0.30
40 days	66.4 ± 14.0	55.9 ± 13.1	73.4 ± 9.4	*p* = 0.64; *p* = 0.35

*Note:* A mixed‐effects model (REML) for one‐way ANOVA with Geisser–Greenhouse correction was performed, followed by Dunnett's test for multiple comparisons.

### 3.4 Histomorphometric Analysis of Peri‐Implant Bone

**FIGURE 7:**
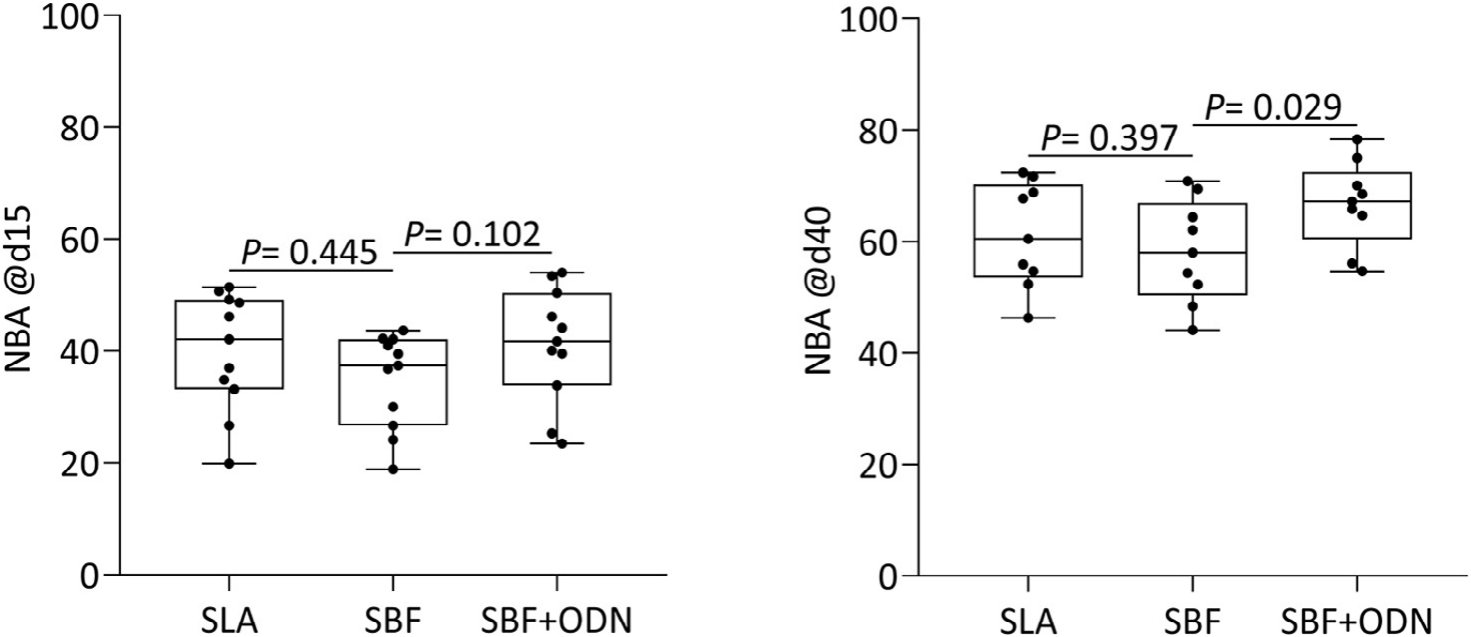
Histomorphometric analysis was conducted immediately adjacent to the implant surface following the indicated healing periods. The graph represents the percentage of new bone area per tissue area (NBA/TA%) for each group: SLA, SBF, and SBF + ODN at Day 15 and Day 40. At 40 days, significant differences were observed comparing SBF and SBF + ODN groups (*p* = 0.029).

**FIGURE 8:**
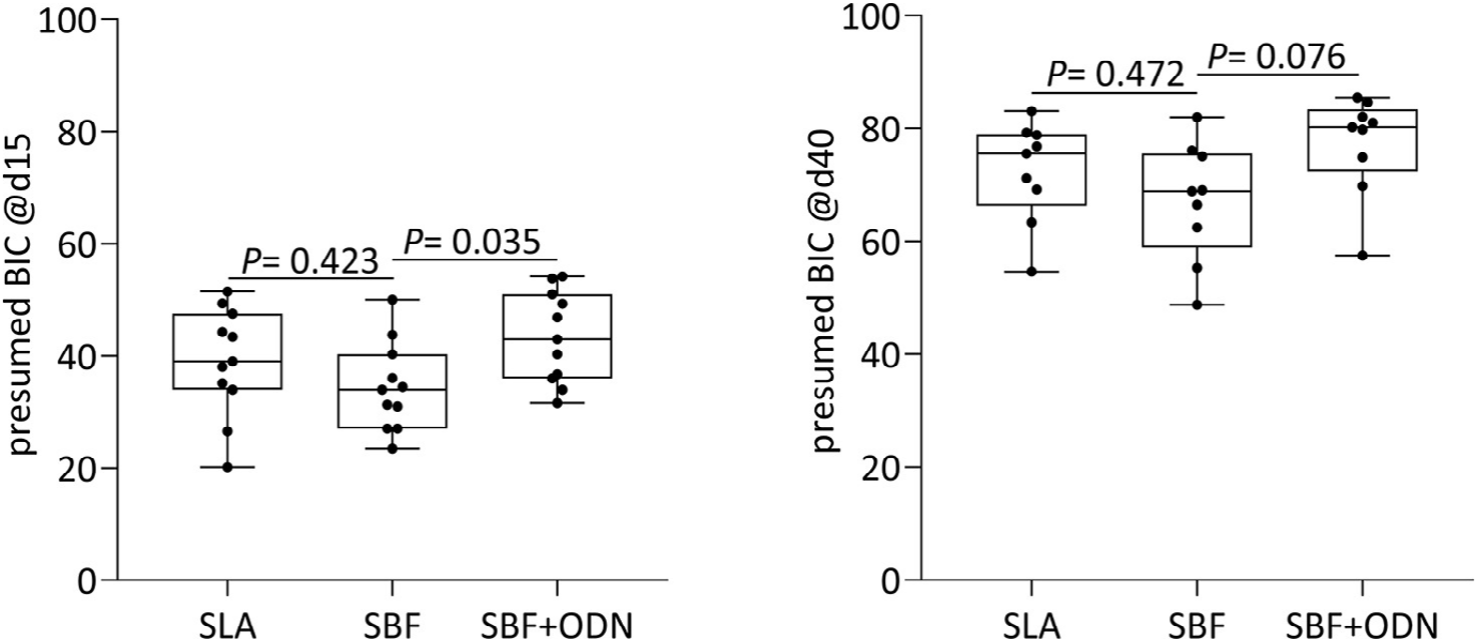
Histomorphometric analysis of the presumed bone‐to‐implant contacts (BIC%) for SLA, SBF, and SBF + ODN at Day 15 and Day 40. A significant statistical difference was identified between the SBF and SBF + ODN groups at both 15 days (*p* = 0.035).

We next performed a histomorphometric analysis of the remaining bone after unscrewing the implants. This analysis showed the obligatory increase of peri‐implant new bone formation between Day 15 and Day 40 (Figures 7 and 8). The histomorphometric analysis further identified a trend for the positive impact of ODN on the peri‐implant new bone formation at Day 15 (*p* = 0.102) and Day 40 (*p* = 0.029). More impressively, ODN coating led to an increase in the pretended BIC on Day 15 (*p* = 0.035) and Day 40 (*p* = 0.076) (Figure 8). In support of the previous analysis, SLA overperformed the SBF‐coated implants based on NBA/TA (Figure 7) and presumed BIC (Figure 8) at Day 15 and Day 40 (Tables 3, 4, 5).

**TABLE 3:** Histomorphometric analysis of new bone area per tissue area (NBA/TA%), the mean, and the standard deviation (±) of the samples for each group: SLA, SBF, and SBF + ODN.

Groups	SLA	SBF	SBF + ODN	*p*
15 days	39.99 ± 10.24	34.78 ± 8.64	41.10 ± 10.28	*p* = 0.445; *p* = 0.102
40 days	61.65 ± 9.39	58.22 ± 9.26	66.71 ± 7.77	*p* = 0.307; *p* = 0.029

*Note:* A mixed‐effects model (REML) for one‐way ANOVA with Geisser–Greenhouse correction was performed, followed by Dunnett's test for multiple comparisons.

**TABLE 4:** Histomorphometric analysis of soft tissues per tissue area (STA/TA%), the mean, and the standard deviation (±) of the samples for each group: SLA, SBF, and SBF + ODN.

Groups	SLA	SBF	SBF + ODN	*p*
15 days	35.53 ± 9.03	39.85 ± 8.34	43.56 ± 11.61	*p* = 0.502; *p* = 0.272
40 days	23.26 ± 11.59	26.25 ± 6.20	21.42 ± 6.19	*p* = 0.324; *p* = 0.390

*Note:* A mixed‐effects model (REML) for one‐way ANOVA with Geisser–Greenhouse correction was performed, followed by Dunnett's test for multiple comparisons.

**TABLE 5:** Histomorphometric analysis of pristine bone per tissue area (PB/TA%), the mean, and the standard deviation (±) of the samples for each group: SLA, SBF, and SBF + ODN.

Groups	SLA	SBF	SBF + ODN	*p*
15 days	23.57 ± 6.59	24.87 ± 5.17	19.76 ± 6.92	*p* = 0.252; *p* = 0.990
40 days	14.92 ± 7.30	15.53 ± 6.49	12.65 ± 4.39	*p* = 0.197; *p* = 0.997

*Note:* A mixed‐effects model (REML) for one‐way ANOVA with Geisser–Greenhouse correction was performed, followed by Dunnett's test for multiple comparisons.

### 3.5 Histological Analysis of Peri‐Implant Bone

**FIGURE 9:**
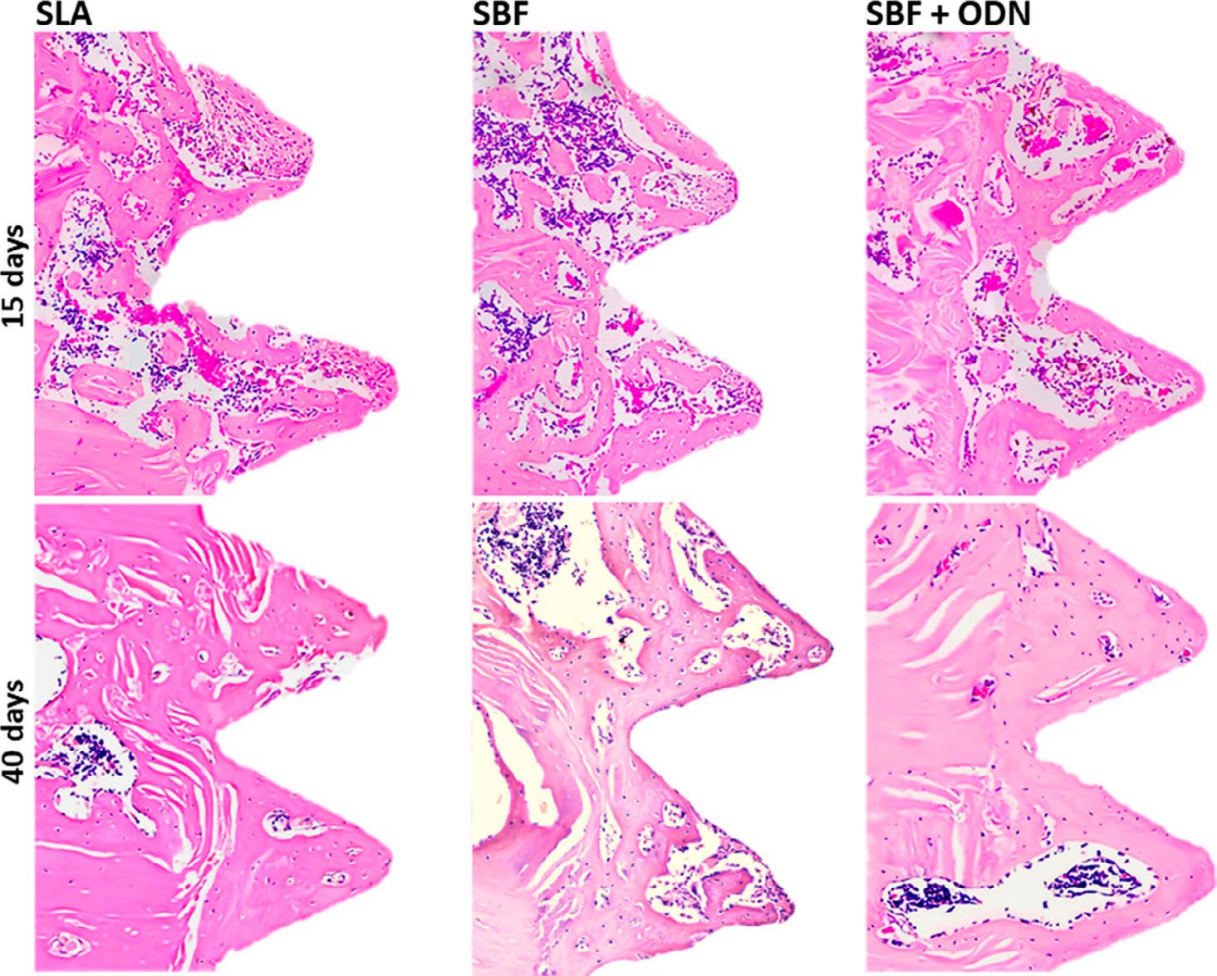
Histological analysis of implant healing was conducted at 15 and 40 days. Representative images depict the medullary compartment confined by the cortical bone edges. Notably, the ODN group exhibited continuous bone integration of the implant at Day 15 and Day 40, with more new bone than the other groups.

Finally, we performed a histological analysis. In general, neither of the samples showed signs of inflammation, suggesting biocompatibility of the SBF coating with the peri‐implant tissues. Peri‐implant bone formation was more evident in the medullary than in the cortical compartment (Figure 9). High magnification of new bone reveals viable osteocytes and areas of osteoblast activity. The direct contact between the newly formed bone and the implant was noticeable, particularly in the SLA and the ODN groups (Figure 9). In addition, we can identify the pristine cortical bone characterized by its lamellar structure.

## 4 Discussion

Implant research has traditionally benefitted from pharmacological strategies aiming to treat osteoporosis, particularly those with an anabolic effect such as the systemic effects of teriparatide (Kuchler et al. 2011). Also, the topical effects of antiresorptive bisphosphonates used for the coating of dental implants (Abtahi et al. 2019; Mokhtari et al. 2024) and orthopedic rods (Tanzer et al. 2005; Zhang et al. 2023) have led to a remarkable increase in early implant stability—however, there are concerns about delayed bone remodeling. Bisphosphonates have the dilemma that while lowering bone resorption, the concomitant bone formation activity is also reduced, at least during bone remodeling (Black et al. 2007; Sims and Martin 2020). This unpleasant situation has prompted the desire for pharmacological strategies aiming to reduce osteoclast activity but allowing them to survive and communicate with the osteoblasts (Sims and Martin 2020)—thus opening a therapeutic window of reduced bone resorption while maintaining bone formation. However, ODN, even though effective in reducing fracture risk, was not approved for osteoporosis therapy because phase III studies revealed severe side effects, including an increased risk of stroke (McClung et al. 2019). These disappointing results have led to a collapse of research strategies based on cathepsin K inhibitors—but while osteoporosis implication requires systemic application of ODN, our concept is based on the topical application of this cathepsin K inhibitor—the coating of implants.

Inspired by the challenges of today's implant concepts to boost the early phase of osseointegration, our research explores the peri‐implant healing of surfaces coated with ODN. Our main finding was that ODN enhances bone formation around implants—this conclusion is grounded on three levels of evidence: biomechanical testing, μCT, and histomorphometric analyses. All results consistently revealed the beneficial effect of ODN in supporting early osseointegration—most of which reached statistical significance, particularly at Day 15 rather than Day 40. The present study consequently supports the original hypothesis that blocking cathepsin K via ODN simultaneously blocks bone resorption while stimulating, or at least not hindering, bone formation during osseointegration. Our observations agree, as far as the functional level is concerned, with studies showing that blocking bone resorption, even with bisphosphonates, is enough to enhance osseointegration in a preclinical setting (Tanzer et al. 2005; Mokhtari et al. 2024). This is in line with previous studies suggesting that bisphosphonates push callus formation in fracture models, though hindering its remodeling (McDonald et al. 2008; Pennypacker et al. 2016). ODN, however, only moderately increased fracture callus but allowed the callus tissue to undergo remodeling (Pennypacker et al. 2016) suggesting a transient activity. Nevertheless, the implication of these divergent observations linked to fracture healing with bisphosphonates and ODN for implant dentistry is unclear. However, they provide a scientific basis for our concept of using a transient antiresorptive principle, based on the inhibition of cathepsin K, for coating dental implants.

Fracture models (McDonald et al. 2008; Pennypacker et al. 2016) along with our observations of implant integration (Tanzer et al. 2005; Mokhtari et al. 2024) suggest that bone formation occurs at early stages of bone healing, regardless of bone resorption. This indicates the absence of the coupling between the osteoclast and osteoblast activities early on, but coupling is central to bone remodeling during the later stages of osseointegration. It can thus be speculated that the beneficial effects of ODN are mainly caused by blocking osteoclast activity (dos Santos Sanches et al. 2025)—rather than requiring its impact on bone remodeling. Nevertheless, even though the cellular mechanism of how ODN acts on osseointegration remains to be answered, the concept is promising as ODN is not incorporated in the new bone matrix; thus, potential adverse effects are unlikely when remodeling becomes relevant. Surprisingly, however, the coating of SLA surface implants with SBF decreased the osseointegration capacity. These results may support previous studies showing that SBF layer thickness, quality, and amount of apatite formation may impair cell adhesion (Kokubo and Takadama 2006; Le Guéhennec et al. 2007). Nevertheless, the reason why SLA surface implants outperform SBF‐coated implants remains speculative, and they are of minor relevance to the present study, which focuses primarily on ODN effects.

Despite the constructive insights provided by our findings, this study has limitations that should be discussed. One limitation is that the actual concentration of ODN in the SBF is not precisely determined, although its activity upon release has been confirmed by bioassay in vitro (dos Santos Sanches et al. 2025). Furthermore, the tibia model does not represent the alveolar bone, and the size of the implants is not ideal for the limited bone marrow space. However, this model enabled bicortical locking, with no signs of inflammation, infection, or implant rejection observed. Our results also confirmed osteointegration, and histological examination revealed that ODN did not cause local adverse effects (Chen et al. 2023). Both observation intervals represent a rather early time point, and longer observation periods might provide additional information. It might also be criticized that the histomorphometric analysis was based on decalcified sections where the implant was removed, rather than on undecalcified histology. Calcified slides would have provided a more precise and detailed visualization of the bone‐implant contact. However, the pretended BIC values were consistent with independent measurements and i.S/TS, collectively establishing a strong scientific basis supporting the overall conclusions. From a clinical perspective, this pilot study provides proof‐of‐principle that cathepsin K inhibitors may support early osseointegration. While the transition from a device to a pharmaceutical, due to the active interference of the coating with cellular function, may hinder potential clinical application, it is important to acknowledge that ODN is not yet approved for any clinical or commercial use worldwide and thus remains an experimental drug. Nevertheless, the concept explored here does not depend exclusively on ODN, since other cathepsin K inhibitors could, in principle, be employed. Importantly, although systemic use of ODN was discontinued because of cardiovascular risk, in the context of a local coating, the risk of generating such adverse effects is practically nil.

In summary, our proof‐of‐principle study offers insights into the potential impact of blocking cathepsin K as a surface coating on the early osseointegration of dental implants. The data suggest that ODN and presumably also other cathepsin K inhibitors hold promise as an adjunct to enhance osseointegration. This preclinical study opens a new chapter exploring ways to boost early osseointegration. Future research should build on this initial approach of targeting cathepsin K, particularly in immediate and early loading protocols.

## Author Contributions

**Natália dos Santos Sanches:** funding acquisition, methodology, writing – review and editing, software, formal analysis, investigation, conceptualization. **Caroline Liberato Marchiolli:** funding acquisition, investigation. **Lara Cristina Cunha Cervantes:** investigation. **Maria Cristina Ruiz Voms Stein:** investigation. **Sara Alves Berton:** investigation. **Estéfany Lopes Lemes do Prado:** investigation. **Carina Kampleitner:** software, formal analysis, validation, visualization. **Francisley Ávila Souza:** investigation, funding acquisition, validation, visualization. **Roberta Okamoto:** investigation, funding acquisition, validation, visualization, conceptualization, data curation. **Reinhard Gruber:** funding acquisition, investigation, writing – original draft, writing – review and editing, validation, visualization, project administration, data curation, supervision, conceptualization, formal analysis, methodology. **Idelmo Rangel Garcia Júnior:** investigation, funding acquisition, project administration, validation, visualization, writing – review and editing, resources, supervision, conceptualization, data curation.

## Disclosure

Implalife, Medical‐Dental Products Industry, Jales, São Paulo, Brazil provided the implants and performed the coating following an unpublished protocol.

## Ethics Statement

The Ethical Committee approved all experimental protocols for Animal Use (CEUA) of the Aracatuba School of Dentistry, UNESP (CEUA 0524‐2021).

## Conflicts of Interest

The authors declare no conflicts of interest.

## Supporting information


**Figure S1:** For implant removal during the paraffin‐embedding process, the specimen was inverted so that the upper portion of the implant faced downward, in contact with the paper mold (A). Once the paraffin had completely solidified (B), the paraffin at the top of the implant was carefully melted without compromising the surrounding bone tissue. The implant was then detached using the square digital key (C) and completely removed (D). The specimen was subsequently re‐immersed in paraffin for 45 min to ensure proper infiltration (E) and repositioned with the implant cavity oriented for the preparation of histological sections (F).

## Data Availability

The data that support the findings of this study are available from the corresponding author upon reasonable request.
