# Supplementary figures and images for: Odanacatib Coating Supports Osseointegration of Implants: A Preclinical Study
Source: Clin Oral Implants Res. 2025 Sep 4;36(12):1640–50. doi: 10.1111/clr.70038 (PMC12669435; doi:10.1111/clr.70038)

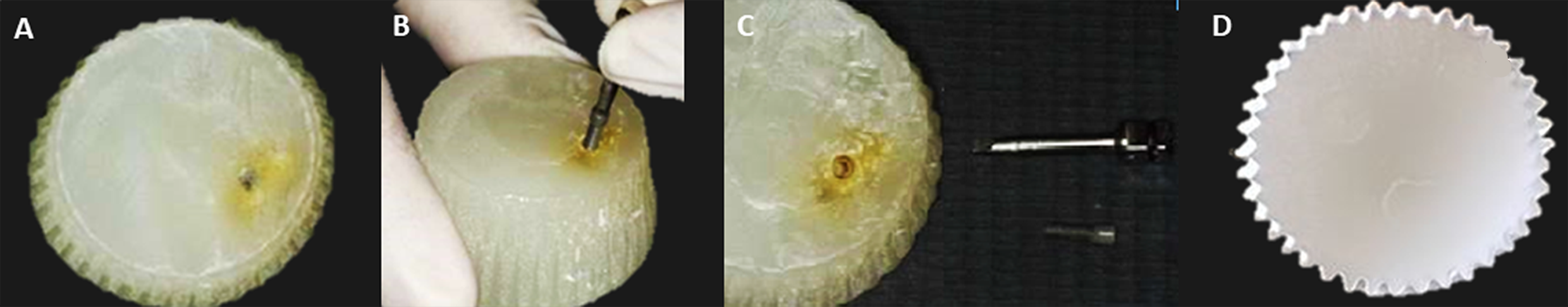

Supplement: Supplementary file 1 — Figure S1: For implant removal during the paraffin‐embedding process, the specimen was inverted so that the upper portion of the implant faced downward, in contact with the paper mold (A). Once the paraffin had completely solidified (B), the paraffin at the top of the implant was carefully melted without compromising the surrounding bone tissue. The implant was then detached using the square digital key (C) and completely removed (D). The specimen was subsequently re‐immersed in paraffin for 45 min to ensure proper infiltration (E) and repositioned with the implant cavity oriented for the preparation of histological sections (F). [file CLR-36-1640-s001.tif]
